# Serum insulin level, disease stage, prostate specific antigen (PSA) and Gleason score in prostate cancer

**DOI:** 10.1038/sj.bjc.6600526

**Published:** 2002-09-23

**Authors:** S Lehrer, E J Diamond, S Stagger, N N Stone, R G Stock

**Affiliations:** Department of Radiation Oncology, Mount Sinai School of Medicine, New York and the Veterans Affairs Medical Center, Bronx, New York, NY 10029, USA; Medicine (Endocrinology), Mount Sinai School of Medicine, New York and the Veterans Affairs Medical Center, Bronx, New York, NY 10029, USA; Urology, Mount Sinai School of Medicine, New York and the Veterans Affairs Medical Center, Bronx, New York, NY 10029, USA

**Keywords:** prostate cancer progression, insulin, growth factors

## Abstract

In the present study, we assessed the relationship of serum insulin levels and three surrogate markers of recurrence, T stage, PSA, and Gleason score, in men with localized prostate cancer. Participants in our study were found through urology and radiation oncology clinics, and all eligible patients were asked to take part. All patients were asymptomatic and had been initially diagnosed on the basis of rising PSA or abnormal physical examination. Histological confirmation of diagnosis was obtained for all subjects. Serum insulin levels were determined by chemoluminescent assay with a standard, commercially available instrument. Patients were divided into three previously defined risk groups: *Low risk*: PSA ⩽10, stage ⩽T2a, or Gleason grade ⩽6. *Medium risk*: 10 <PSA ⩽15, Gleason 7 or stage T2b. *High risk*: Gleason >7, tumour in seminal vesicle biopsy, PSA >15 or stage T2c or T3. One hundred and sixty-three men with prostate cancer were studied. There was a significant increase in serum insulin with risk group (*P*=0.003, one way anova). Tukey's multiple range test showed that the insulin levels of high risk patients were significantly higher than the insulin levels of medium and low risk patients (*P*=0.05) but the insulin levels of medium and low risk patients were not significantly different from one another. Multivariate linear regression, with insulin as the dependent variable, Gleason score, PSA, and T stage (T1, T2, T3) as the independent variables, was significant overall (*P*<0.001, *r*^2^=0.120). Increased T stage was independently correlated with increased serum insulin levels (*P*<0.001). Gleason score was negatively, insignificantly correlated with serum insulin level (*P*=0.059). The positive correlation of PSA and insulin level was not significant (*P*=0.097). To assure normal distribution of insulin and PSA values, the regression was repeated with log (insulin) as the dependent variable, log (PSA), T stage (T1, T2, T3), and Gleason score as independent variables. The regression was significant overall (*P*=0.002, *r*^2^ =0.095). Increased T stage was independently correlated with increased log (insulin level) (*P*=0.026). Gleason score was negatively, insignificantly correlated with log (insulin) level (*P*=0.728). The positive correlation of log (PSA) and log (insulin) levels was significant (*P*=0.010). The relationship between increased insulin level and advanced tumour stage in prostate cancer we describe here is biologically quite plausible, since insulin is a growth factor. Further studies may document whether serum insulin levels might be a useful biomarker of prostate cancer stage.

*British Journal of Cancer* (2002) **87**, 726–728. doi:10.1038/sj.bjc.6600526 www.bjcancer.com

© 2002 Cancer Research UK

## 

PSA, Gleason score, and stage at diagnosis are currently the most reliable markers of prostate cancer prognosis and tumour aggressiveness (Oesterling *et al*, 1997). But urologists are actively seeking additional biomarkers.

Many prostate cancers are quite indolent and may never cause a problem, but it is now impossible to identify such tumours with certainty. With more and better biomarkers, some older men might be spared the rigors of radiation therapy and/or surgery, and their complications.

Elevated insulin levels are associated with increased risk of prostate cancer (Hsing *et al*, 2001) and increased risk of metastatic disease in women with breast cancer (del Giudice *et al*, 1998; Goodwin *et al*, 2000). Moreover, insulin is necessary for the growth of prostate cancer cells in culture. For example, a special serum-free defined medium that can support short-term, long-term, and clonal growth of the human prostatic carcinoma cell lines LNCaP, DU 145, PC-3, and ALVA-31 must contain insulin (Hedlund and Miller, 1994).

In a previous study (Lehrer *et al*, 2002), we measured insulin levels in men with prostate cancer. We found that increased serum insulin levels were associated with increased risk of recurrence. We determined risk of recurrence by dividing the patients into previously defined risk groups (Stock *et al*, 2000), consisting of three parameters: prostate specific antigen (PSA) level, tumour stage, and Gleason score. We have now collected a larger patient sample and have performed multivariate analysis to ascertain which of the three parameters are most related to insulin level.

## METHODS

Participants in our study were found through urology and radiation oncology clinics, and all eligible patients were asked to take part. All patients had been initially diagnosed on the basis of rising PSA or abnormal physical examination. Histological confirmation of diagnosis was obtained for all subjects. All participants gave informed consent. All staging was clinical, because almost all the patients were to receive I-125 seed implant.

We studied men referred for treatment of localized prostate cancer. In our treatment protocol, patients are divided into three risk groups (Stock *et al*, 2000).

### Low risk

PSA ⩽10, stage ⩽T2a, or Gleason grade ⩽6. These patients are treated with a radioactive implant.

### Medium risk

10 <PSA ⩽15, Gleason 7 or stage T2b. These patients are treated with 3 months combined hormonal therapy followed by an implant.

### High risk

Gleason >7, tumour in seminal vesicle biopsy, PSA >15 or stage T2c or T3. These patients are treated with 3 months' combined hormonal therapy, an implant, and after 2 months' break 6000 rads with external beam radiotherapy.

Serum insulin levels were determined by chemoluminescent assay with a standard, commercially available instrument (Immulite Diagnostic Products Corporation, Los Angeles, CA, USA).

## RESULTS

Patients studied: 163; 73 were low risk, 22 medium risk, and 68 high risk. The youngest was 46, the oldest 88, average age 67±8.2 (mean ±s.d.).

**Figure 1:**
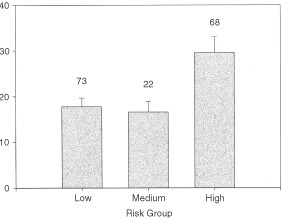
Serum insulin (μIu ml^−1^) levels of men with prostate cancer. Number of cases in each risk group is above the error bar. There was significant variation of insulin (*P*=0.003, one way anova). Tukey's multiple range test showed that the insulin levels of high risk patients were significantly higher than the insulin levels of medium and low risk patients (*P*=0.05) but the insulin levels of medium and low risk patients were not significantly different from one another. (The normal insulin range in our laboratory is 6–27 μIu ml^−1^).

There was a significant increase in serum insulin with risk group (*P*= 0.003, one-way anova, Figure 1

). Tukey's multiple range test showed that the insulin levels of high risk patients were significantly higher than the insulin levels of medium and low risk patients (*P*=0.05) but the insulin levels of medium and low risk patients were not significantly different from one another.

Multivariate linear regression, with insulin as the dependent variable, Gleason score, PSA, and T stage (T1, T2, T3) as the independent variables, was significant overall (*P*<0.001, *r*^2^=0.120). Increased T stage was independently correlated with increased serum insulin level (*P*<0.001). Gleason score was negatively, insignificantly correlated with serum insulin level (*P*=0.059). The positive correlation of PSA and insulin level was not significant (*P*=0.097).

To assure normal distribution of insulin and PSA values, the regression was repeated with log (insulin) as the dependent variable, log (PSA), T stage (T1, T2, T3), and Gleason score as independent variables. The regression was significant overall (*P*=0.002, *r*^2^=0.095). Increased T stage was independently correlated with increased log (insulin level) (*P*=0.026). Gleason score was negatively, insignificantly correlated with log (insulin) level (*P*=0.728). The positive correlation of log (PSA) and log (insulin) levels was significant (*P*=0.010).

## DISCUSSION

One possible weakness in our study is the fact that serum insulin levels were measured in the three treatment groups after therapy had been completed. Therefore, it would be worthwhile in future studies to measure serum insulin levels both before and after therapy, in order to correct for possible error caused by the differences in treatment delivered to the three risk groups. Another possible weakness is the relatively smaller number of cases in the intermediate risk group, when compared to the low and high risk groups. Further larger studies should be done to eliminate this weakness.

Data from epidemiological and biological research implicate insulin-like growth factors I and II (IGF-I and IGF-II) in the regulation of prostate epithelial cell proliferation, and in the pathophysiology of prostate cancer (Pollak *et al*, 1998). But there has been little investigation into the role insulin itself plays in prostate cancer. Isolated epithelial cells of rat ventral prostate have insulin receptors, and fasting increases their concentration (Carmena *et al*, 1986). But PA-III rat prostate adenocarcinoma cells have no insulin receptor, though they do have specific binding sites for IGF-I and II (Polychronakos *et al*, 1991).

One large epidemiologic study found an equivocal relationship between diabetes mellitus and prostate cancer. In hundreds of thousands of male respondents, the 1959–1972 Cancer Prevention Study explored whether men with diabetes were more likely to develop prostate cancer during a 13-year follow-up than men without diabetes. After adjustment for factors associated with prostate cancer in previous studies, little association was found between diabetes at baseline and prostate cancer incidence. Men who had diabetes mellitus for five or more years, however, had a higher incidence of prostate cancer than did men without diabetes. But among all study participants who were diagnosed with prostate cancer, men with diabetes were only slightly more likely to die from prostate cancer than were men without diabetes (Will *et al*, 1999).

The relationship between increased insulin level and advanced tumour stage in prostate cancer we describe here is biologically quite plausible, since insulin is a growth factor. Further studies may document whether serum insulin level might be a useful biomarker of prostate cancer stage.
